# A qualitative assessment of factors influencing acceptance of a new rotavirus vaccine among health care providers and consumers

**DOI:** 10.1186/1471-2431-7-32

**Published:** 2007-10-18

**Authors:** Manish M Patel, Alan P Janssen, Richard R Tardif, Mark Herring, Umesh D Parashar

**Affiliations:** 1 Centers for Disease Control and Prevention, National Center for Immunizations and Respiratory Diseases, Division of Viral Diseases, Epidemiology Branch, Atlanta, Georgia, USA; 2 Centers for Disease Control and Prevention, National Center for Immunizations and Respiratory Diseases, Immunizations Services Division Atlanta, Georgia, USA; 3 Oak Ridge Institute for Science and Education, Oak Ridge, Tennessee, USA; 4 Market Directions, Kansas City, Missouri, USA; 5 1600 Clifton Road, MS A-47, Atlanta, GA 30329, USA

## Abstract

**Background:**

In 2006, a new rotavirus vaccine (RotaTeq) was licensed in the US and recommended for routine immunization of all US infants. Because a previously licensed vaccine (Rotashield) was withdrawn from the US for safety concerns, identifying barriers to uptake of RotaTeq will help develop strategies to broaden vaccine coverage.

**Methods:**

We explored beliefs and attitudes of parents (n = 57) and providers (n = 10) towards rotavirus disease and vaccines through a qualitative assessment using focus groups and in-depth interviews.

**Results:**

All physicians were familiar with safety concerns about rotavirus vaccines, but felt reassured by RotaTeq's safety profile. When asked about likelihood of using RotaTeq on a scale of one to seven (1 = "absolutely not;" 7 = "absolutely yes") the mean score was 5 (range = 3–6). Physicians expressed a high likelihood of adopting RotaTeq, particularly if recommended by their professional organizations and expressed specific interest in post-marketing safety data. Similarly, consumers found the RotaTeq safety profile to be favorable and would rely on their physician's recommendation for vaccination. However, when asked to rank likelihood of having their child vaccinated against rotavirus (1 = "definitely not get;" 7 = "definitely get"), 29% ranked 1 or 2, 36% 3 or 4, and 35% 5 to 7.

**Conclusion:**

Our qualitative assessment provides complementary data to recent quantitative surveys and suggests that physicians and parents are likely to adopt the newly licensed rotavirus vaccine. Increasing parental awareness of the rotavirus disease burden and providing physicians with timely post-marketing surveillance data will be integral to a successful vaccination program.

## Background

In February 2006, a new rotavirus vaccine (RotaTeq, Merck) was licensed by the US Food and Drug Administration (FDA) and was recommended by the Advisory Committee on Immunization Practices (ACIP) for routine immunization of all US infants at 2, 4, and 6 months of age[1]. This vaccine, which in clinical trials demonstrated an efficacy of 74% against any rotavirus disease and 98% against severe rotavirus disease,[2] holds great promise for the prevention of the more than 2.7 million episodes of diarrhea, 400,000 outpatient office visits, and 55,000–70,000 hospitalizations per year attributable to rotavirus in US children <5 years of age [3]. Identifying barriers to uptake of this vaccine will help policy makers and public health officials develop strategies to achieve high immunization rates for optimum disease prevention efforts.

A well-recognized potential barrier to acceptance of RotaTeq is the association of a previous rotavirus vaccine (Rotashield, Wyeth Laboratories) with intussusception, a potentially life-threatening intestinal blockage [4-6]. Because of this adverse event, Rotashield was withdrawn from the market less than a year after its introduction in August 1998[4,5]. Although RotaTeq has not been associated with intussusception or other serious adverse events in large pre-licensure clinical trials [1,2], the abrupt withdrawal of Rotashield garnered considerable nationwide attention and could affect adoption of the vaccine by parents and healthcare workers. In a national survey of US pediatricians, possible reluctance of parents to accept the new vaccine because of the safety issues associated with Rotashield was indeed identified as a potential major barrier to acceptance [6,7]. However, a limitation of structured quantitative surveys is that the questions may not have allowed for a complete disclosure of all issues and beliefs with regard to rotavirus vaccination [8]. In addition, to our knowledge, no direct assessment of the attitudes and beliefs of parents towards the rotavirus vaccine has been conducted.

To directly explore the beliefs and attitudes of parents of US children towards rotavirus vaccines and to gain further insight into the providers' concerns, we conducted a qualitative study of prospective providers and consumers of the rotavirus vaccine. Our objectives were to identify issues and concerns related to rotavirus disease and vaccinations among both providers and consumers so that we can develop strategies for improving adoption of the new rotavirus vaccine.

## Methods

### Sample and data collection

Our study sample consisted of two groups: healthcare providers and consumers. Study participants went through the same study process at two different locations: Sunnyvale, California and Kansas City, Missouri.

#### Providers

Physician participants were recruited by market research firms that were asked to contact actively practicing, non-military, non-institutional physicians who practice in their communities. Participants were further screened, limiting selection to board-certified or board-eligible pediatricians or family medicine physicians who typically administer 5 or more immunizations each week. At each of the two study sites, we enrolled a minimum of one pediatrician and one family medicine practitioner who had been in practice for 10 years or longer to ensure participation of a provider who was practicing during the period when Rotashield was withdrawn. We excluded participants if an immediate family member worked in advertising, public relations, marketing research, or the media.

Because our primary interest regarding providers was their personal experience and perspective on rotavirus disease and vaccines (i.e., Rotashield and RotaTeq), we choose to conduct in-depth interviews as the data collection method for this group. A moderator with over 25 years of experience conducted individual 60-minute interviews using a guide that facilitated the discussion. In general, after introduction of the project, the moderator asked each provider to discuss his/her treatment algorithm for a typical child who presents to his/her clinic with diarrhea. Subsequently, the provider was prompted to discuss issues pertaining to the previous Rotashield vaccine. The moderator then asked the provider to review the FDA package insert with information on the recently introduced RotaTeq vaccine. Issues relating to the safety, acceptability, and future utilization of RotaTeq were explored. Lastly, each provider reviewed the rotavirus Vaccine Information Statement, a CDC public information sheet on rotavirus disease and vaccines, and was asked to render his/her opinion about patient-education materials.

#### Consumers

Consumer groups were recruited from a panel of persons that had previously indicated an interest in participating in focus groups. For this group, we recruited women older than 18 years who reported themselves as "likely or very likely" to give birth to a child within 4 years and expressed intent to have their child receive routine vaccinations against childhood diseases. We excluded women who were employed in the healthcare field or in the media or who had participated in any market studies within the previous six months.

We convened four 90-minute focus groups in each study site. Women were categorized according to their child-rearing (CR) experience (experience raising > one child to age 6) and their level of concern (LOC) regarding the safety of childhood vaccinations (i.e., high = self-reported as being "very" or "moderately" concerned; low = self-reported as having "no" or "a little" concern). The four focus groups were a combination of women in these two categories (e.g., group 1: LOC = high and CR = yes). We ensured that each group consisted of > 2 participants with less than a Bachelor's degree of formal education and > 2 participants with a Bachelor's degree or more of formal education.

After introduction to the project, each focus group began with a discussion of the general childhood health concerns that the participants regarded to be important. The discussion subsequently was directed to diarrhea and rotavirus disease and the participants' perception of the importance of these diseases. Participants were asked to rank the seriousness of rotavirus disease on a scale of one to seven, with one being "not serious at all" and seven being "very serious." Lastly, participants reviewed the rotavirus Vaccine Information Statement and discussed their knowledge, issues, concerns, and attitudes towards rotavirus vaccines. On a scale of one to seven, where one was "definitely not get" and seven was "definitely get," participants were asked to rank their likelihood of having their child vaccinated against rotavirus. To facilitate and focus the discussion, throughout the session, the moderator used flipcharts and printed handouts relevant to rotavirus disease.

All provider interviews and consumer focus group sessions were audio-taped and conducted in English during March 2006, by independent moderators from a market research facility (Market Directions, Inc.). Study investigators and data recorders were present behind a one-way mirror and were available to answer questions at the end of each session or interview. All participants were provided a moderate reimbursement for their time. The study was reviewed and approved by CDC's Institutional Review Board.

## Results

Overall, we enrolled 10 providers and 57 consumers in our study with varying levels of education, training, and experience; diverse racial and ethnic backgrounds; and broad levels of concern about vaccine safety issues (Tables 1 and 2). Below, we present the data stratified according to the two groups of study participants.

**Table 1:** Characteristics of providers participating in the study, by study location

*Characteristics*	*Study site*	
	
	Sunnyvale, California (N = 5)	Kansas City, Missouri (N = 5)
Specialty		
Pediatrics	3	3
Family medicine	2	2
Race/ethnicity		
White	3	4
Asian American	2	1
Gender		
Male	3	4
Female	2	1
Years in practice		
<10 years	1	3
≥10 years	4	2

**Table 2:** Characteristics of consumers participating in the study, by study location

Characteristics	*Study site*	
	
	Sunnyvale, California (N = 26)	Kansas City, Missouri (31)
Education		
High school or less	5	10
More than high school	21	21
		
Race/ethnicity		
White	9	20
Black	5	9
Asian American	3	0
Hispanic	6	1
Other	3	1
		
Focus group strata*		
LOC high & CR yes	8	6
LOC high & CR no	7	9
LOC low & CR yes	7	8
LOC low & CR no	5	8

*LOC denotes level of concern about the safety of childhood vaccines (i.e., "high" or "low") and CR denotes child-rearing experience raising > 1 child to age 6 (i.e., "yes" or "no").

### Providers

All providers recognized the importance and impact of severe dehydration from diarrheal disease and reported a similar treatment algorithm that depended on the severity of the dehydration. With regard to the vaccines, several consistent themes emerged from the in-depth provider interviews (Table 3) that can be categorized as: (1) vaccine perception; (2) future utilization of vaccine; and (3) vaccine informational material.

**Table 3:** Consistently emerging themes from the provider interviews and the consumer focus groups – Sunnyvale, CA and Kansas City, MO.

***Provider interviews:***

• All physicians were familiar with Rotashield.
• Vaccine regarded as having an excellent safety and efficacy profile.
• Providers accurately predicted parental perception of vaccine safety and efficacy.
• Physicians likely to use RotaTeq if recommended by the Advisory Committee on Immunization Practices (ACIP).
• CDC's Vaccine Information Sheet noted to be accurate and useful for parents.
• All physicians reported a consistent treatment algorithm for diarrhea.
• Expressed interest in post-licensure safety and effectiveness data.

**Consumer focus groups:**

• Overall, rotavirus disease was not perceived to be a high-priority childhood health issue.
• Vaccine found to be acceptable and perceived in a positive light
• Vaccine concerns included: administration of a live-virus, "newness" of the vaccine, potential for adverse events, and narrow window of opportunity to vaccinate.
• Noted desire for more information with regard to rotavirus disease and vaccines.
• 16% of the consumers claimed that their child would "definitely not get" the vaccine.
• Physician's recommendation to vaccinate their child would be persuasive.

#### Vaccine perception

Overall, all physicians were familiar with Rotashield and the new vaccine, RotaTeq. Participants anticipated that Rotashield's intussusception history would contribute to the general anti-vaccine sentiment that is expressed by a small minority of their patients. However, all physicians accepted RotaTeq as having an excellent safety and efficacy profile and welcomed the incorporation of the vaccine into the routine immunization schedule. With regard to parental acceptance, physicians predicted that the vaccine's safety and efficacy would be well-perceived by the parents, but they also predicted that the "live" nature of the vaccine and the history of Rotashield would be a potential barrier to acceptance for some parents.

#### Future utilization of vaccine

When asked to rate their likelihood of using the new vaccine on a scale of one to seven, with one being "absolutely not" and seven being "absolutely yes," the mean score was 5 (range = 3 to 6). Nonetheless, all providers remarked that they would recommend the rotavirus vaccine to parents if recommended for use by the ACIP and their respective professional organizations (i.e., Academy of Family Practice and American Academy of Pediatrics). The general sentiment was that once the vaccine became part of the routine immunization schedule, they would spend very little time discussing or promoting the individual vaccine and the vaccine would likely be administered to all eligible children. A majority of the physicians also relayed an unaided response voicing a keen interest in following studies that continue to shed light on post-licensure effectiveness and safety of the vaccine.

#### Vaccine information material

All physicians reviewed the CDC's Vaccine Information Statement and found the content to be comprehensive, clear, and useful for the parents.

When asked, none recommended any specific modifications for the Vaccine Information Statement.

### Consumers

Among prospective consumers of the vaccine, a lack of awareness about rotavirus disease and need for more information about the disease and the vaccine was evident. General themes emerging from the focus groups can be classified according to: (1) rotavirus knowledge; (2) rotavirus vaccines; and (3) informational material.

#### Rotavirus knowledge

**Figure 1:**
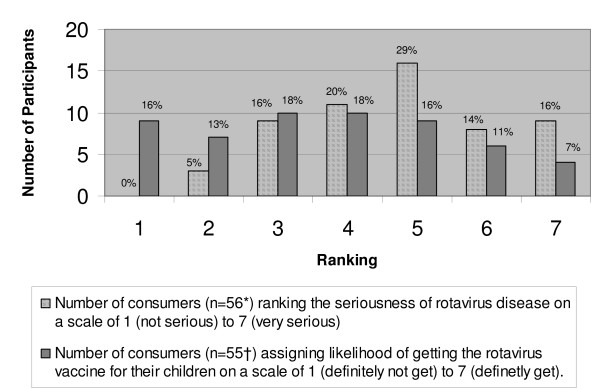
**Consumer perception of the seriousness of rotavirus disease and likelihood of getting the vaccine for their children**. *Data missing on 1 participant. †Data missing for 2 participants.

Prior to receiving printed informational material describing rotavirus disease and prevention through vaccination, most participants were not aware of the public health impact of rotavirus or its potential for causing severe disease and death. Participants did not rate the disease to be a high-priority health issue for children. In Sunnyvale, most participants reported never having heard of rotavirus prior to the focus group. However, in Kansas City, where a seasonal peak in rotavirus disease was ongoing at the time of the focus group, an increased awareness of rotavirus disease was noted among the participants. Perceived seriousness of rotavirus diseases was somewhat greater in Sunnyvale among women without child-rearing experience. Interestingly, once participants reviewed the Vaccine Information Statement, most considered rotavirus disease to be a moderately serious condition. Based on a scale of 1 ("not at all serious") to 7 ("very serious"), 59% consider the seriousness of rotavirus disease to be very serious (range 5 to 7), while 36% consider the disease to be moderate (range = 3 to 4) and only 5% consider it to be not at all serious (range = 1 to 2) (Figure 1).

#### Rotavirus vaccines

Upon reviewing the Vaccine Information Statement, consumers generally deemed the vaccine to be acceptable and commented positively on several aspects of the vaccine such as the oral formulation, high efficacy in preventing severe disease, administration of the vaccine during routinely scheduled physician visits, and overall safety of the vaccine. However, when asked to rank the likelihood of having their child vaccinated against rotavirus from 1 ("definitely not get") to 7 ("definitely get"), 29% ranked between 1 to 2, 36% between 3 to 4, and 35% between 5 to 7 (Figure 1). Generally, those participants with previous child-rearing experience were less concerned about vaccinations. However, two respondents expressed increased level of awareness with regard to vaccine safety issues with their second child. Commonly expressed concerns about the vaccine included the administration of a live-vaccine to "young, vulnerable" infants, the newness of the vaccine, potential for adverse events as more data becomes available, and the narrow window of age when the vaccine is recommended. Despite these concerns, most consumers reported that they would rely on their physician's recommendation on whether their child should receive the rotavirus vaccine.

#### Informational material

In general, the rotavirus Vaccine Information Statement generated interest and raised awareness about rotavirus disease among the consumers, but it also raised more questions about the biology and epidemiology of rotavirus disease and reasons for not vaccinating beyond six months of age. Consumers expressed a desire for more information about rotavirus disease and identified the Internet as a primary source of obtaining health information. Study participants expressed preference for marketing websites such as WebMD.com and babycenter.com and few recognized CDC as a source of source of health information for the public.

## Discussion

Our qualitative assessment using focus groups and in-depth interviews of prospective rotavirus vaccine consumers and providers, respectively, provided several valuable insights about potential barriers to immunizing US children against rotavirus and identified issues that need to be better assessed through quantitative surveys. Most providers appreciated the health burden of rotavirus disease and the need for a vaccine. While they were aware of the safety issues with the previous rotavirus vaccine, they felt reassured by the safety profile of the new rotavirus vaccine and expressed a high likelihood of adopting the new vaccine, particularly if it were recommended by their professional organizations. In addition, most providers expressed specific interest in following results of the post-licensure vaccine effectiveness studies and data from adverse events monitoring. This suggests that post-marketing surveillance for effectiveness and safety should be a high priority for the public health and research community and the results should be rapidly communicated to providers.

Unlike physicians, however, women who participated in these focus groups were considerably less aware of the health burden of rotavirus. In our study, many focus group participants, particularly those living in Sunnyvale, were completely unaware about rotavirus disease. Although these women initially did not perceive rotavirus diarrhea to be a high-priority health issue, a majority considered rotavirus to be a moderate to severe illness after reading the Vaccine Information Statement. Furthermore, they asked many pertinent questions about the epidemiology of rotavirus and risk factors for severe disease. Lack of disease awareness may be one barrier to acceptance of the new vaccine, particularly if parents perceive the risks of vaccination to be greater [9,10]. Nevertheless our data suggest that regardless of rotavirus knowledge, if the provider recommends vaccination, parents would likely accept the vaccine. Because providers stated that they would follow recommendations from ACIP and their professional organizations, dissemination of rotavirus vaccine and disease information through these organizations could be important to increase vaccine uptake. However, further studies would be necessary to assess whether these interventions actually contribute towards increasing vaccination coverage rates.

Further exploration for the reasons for the lack of consumer familiarity with rotavirus disease suggests that traditional media venues used by public health and medical communities to disseminate information may not be reaching the target audience. In our study, most participants did not use or recognize the CDC portal as a source of health information for the public, but reported using online sources of health information that are managed by private companies. Utilizing such broader channels to disseminate health information may be necessary to raise the public awareness about rotavirus disease. Interestingly, Kansas City residents were more aware of rotavirus disease than those living in Sunnyvale, possibly because, in Kansas City, the study was conducted during the peak rotavirus season.

Some limitations should be considered in interpretation of the findings. First, we interviewed relatively few physicians and consumers from two selected geographic locations. The unique characteristics of our study sample may limit the application of these findings to a broader population. For example, most of the participants in Sunnyvale were unaware of rotavirus disease prior to their involvement in the focus group. Secondly, qualitative surveys can be limited by the subjective interpretation of the data and should be verified through quantitative surveys of a large and representative sample [8,11]. We observed, however, a consistency in the responses from both providers and participants which suggests that these findings may be broadly applicable. Furthermore, the responses from the providers are consistent with those observed in larger national surveys of providers [6,7]. Our qualitative survey methodology was also useful in identifying important themes about rotavirus disease that were complementary to findings of quantitative surveys and thus provide a comprehensive assessment of barriers to rotavirus vaccination.

In conclusion, the findings of our survey indicate that US providers are aware of the health burden of rotavirus and are likely to adopt the new rotavirus vaccine, even though they were aware of the safety concerns with the earlier rotavirus vaccine. Parents were substantially less aware of rotavirus disease, although most expressed that they would get their child vaccinated if their physician recommended it.

## Competing interests

The author(s) declare that they have no competing interests.

## Authors' contributions

AJ, RT, and MH conceived, designed, and implemented the project. UP participated in the design of the study, interpretation of the data, and drafting of the manuscript. MP assisted with the interpretation of the data and drafted the manuscript. All authors read and approved the final manuscript.

## Pre-publication history

The pre-publication history for this paper can be accessed here:


